# Effects of temperature and moisture fluctuations for suitable use of raw-crushed wind-turbine blade in concrete

**DOI:** 10.1007/s11356-024-33720-0

**Published:** 2024-05-24

**Authors:** Víctor Revilla-Cuesta, Nerea Hurtado-Alonso, Javier Manso-Morato, Roberto Serrano-López, Juan M. Manso

**Affiliations:** 1 Department of Civil Engineering, Escuela Politécnica Superior, University of Burgos, C/ Villadiego S/N, 09001 Burgos, Spain; 2 Department of Construction, Escuela Politécnica Superior, University of Burgos, C/ Villadiego S/N, 09001 Burgos, Spain

**Keywords:** Raw-crushed wind-turbine blade, Concrete, Moist/dry test, Alternating-sign temperatures, Thermal shock, Multiple-regression statistical prediction

## Abstract

Raw-crushed wind-turbine blade (RCWTB), a waste from the recycling of wind-turbine blades, is used as a raw material in concrete in this research. It contains not only fiberglass-composite fibers that bridge the cementitious matrix but also polyurethane and balsa-wood particles. Therefore, concrete containing RCWTB can be notably affected by moisture and temperature fluctuations and by exposure to high temperatures. In this research, the performance of five concrete mixes with 0.0%, 1.5%, 3.0%, 4.5%, and 6.0% RCWTB, respectively, is studied under moist/dry, alternating-sign-temperature-shock, and high-temperature-shock tests. Two damage mechanisms of RCWTB within concrete were found through these tests: on the one hand, micro-cracking of the cementitious matrix, which was verified by microscopic analyses and was dependent on concrete porosity; on the other, damage and degradation of the RCWTB components, as the polyurethane melted, and the balsa-wood particles burned. Both phenomena led to larger remaining-strain levels and reduced concrete compressive strength by up to 25% under temperature and humidity variations, although the bridging effect of the fiberglass-composite fibers was effective when adding RCWTB amounts higher than 3.0%. The compressive-strength loss after the high-temperature-shock test increased with the RCWTB content, reaching maximum values of 8% after an exposure time of 7 days. Statistical analyses revealed that effect of the RCA amount in the concrete was conditioned by the exposure times in all the tests. The accurate definition of those times is therefore key to set an RCWTB content in concrete that ensures its suitable behavior under the environmental conditions analyzed.

## Introduction

**Fig. 1:**
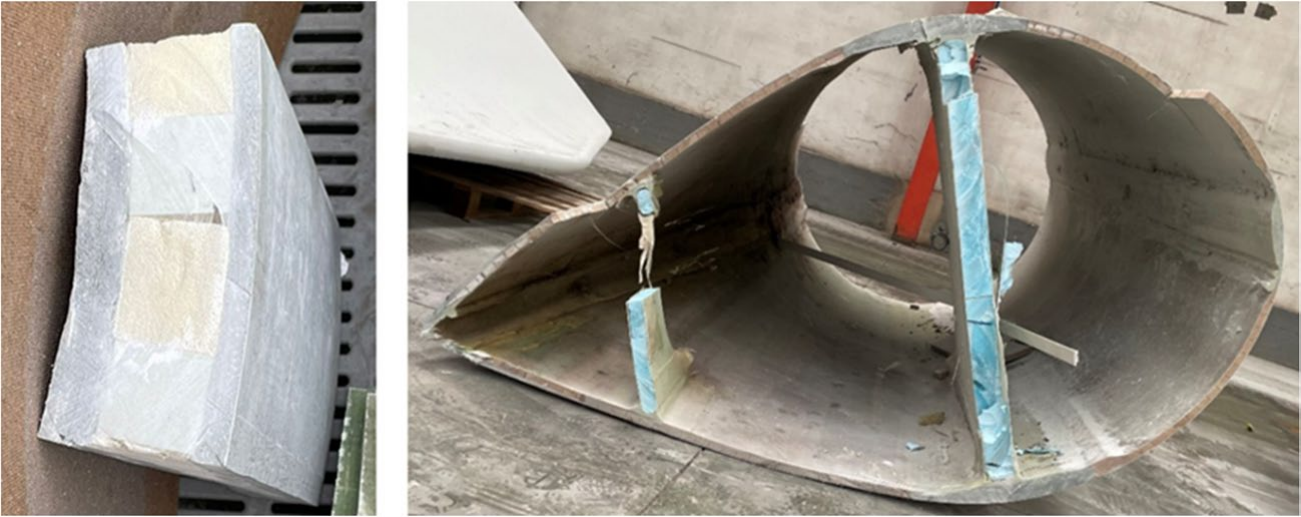
Sandwich panel (left) and section (right) of a decommissioned wind-turbine blade

A wind-turbine blade is aerodynamically designed to balance lightness and structural strength (Özkan and Genç 2023). Therefore, they combine materials with mechanical strength with other light-weight materials (Haselbach et al. 2022), which causes that their composition is quite complex (Joustra et al. 2021). Glass fiber-reinforced polymer (GFRP) composite is commonly used as the main blade material (Mallesh et al. 2019), as it has a high tensile strength (Ingersoll and Ning 2020). However, its high density is often mitigated with lighter materials such as balsa wood (Pan et al. 2018), which has an appearance similar to cork and a density of 0.25–0.35 kg/dm^3^ (Jang and Kang 2022). Both materials are usually arranged in sandwich panels or one after the other on the blade wall, as shown in Fig. 1. In addition, polyurethane is often added, which forms the inner stiffeners of the blade (Fig. 1 right) and is sometimes used in joining elements (Marín and Graciani 2022). Finally, a protective exterior gel coating is applied to the blade (Zhang et al. 2021).

In view of the above, it is clear that wind-turbine blade designs were never conceived with recycling in mind, due to the very varied materials within one blade (Gennitsaris et al. 2023; Ozturk and Karipoglu 2023). So, today, one of the great challenges of the wind-energy sector is to address the issue of recycling these elements (Sommer and Walther 2021), whose urgency is pressing, as the first wind farms installed will reach the end of their useful life in the coming years (Liu and Barlow 2017; AEE 2022). Thus, varied solutions have been offered to give a second life to the materials that make up wind-turbine blades. First, blade components can be separated through chemical formulations such as solvolysis or pyrolysis (Fonte and Xydis 2021). Second, mechanical separation of blade components using cutting processes can also be conducted (Yazdanbakhsh et al. 2018). A third option involves crushing and then sieving to separate the blade materials (G. T. Xu et al. 2022a, b).

One potential usage of the raw materials recovered after these treatments is the production of concrete (Baturkin et al. 2022). Recycled fibers from chemical treatments of GFRP composite could be used for the manufacture of fiber-reinforced concrete (Rodsin et al. 2022), thereby providing the concrete with a slightly higher load-bearing capacity (Barris et al. 2023). The GFRP composite separated from the other components after a cutting process can be machined to obtain aggregate-like particles with which to reduce natural-aggregate consumption (Yazdanbakhsh et al. 2018). Finally, the crushed GFRP composite, separated after sieving out all other components, can also be successfully added to the concrete in the form of fibers (G. T. Xu et al. 2022a, b) or powder (Pławecka et al. 2021).

Nevertheless, all these processes have drawbacks. Chemical processes are energy intensive and lead to greenhouse gas emissions (Kawajiri and Kobayashi 2022; Al-Fatesh et al. 2023). Mechanical processes only recover the recycled GFRP composite, so the question of what to do with all the other materials remains unresolved (Liu and Barlow 2017). In this study, the selected approach was to crush the entire wind-turbine blade without component separation to produce raw-crushed wind-turbine blade (RCWTB). This material, composed of GFRP-composite fibers and small particles of balsa wood and polyurethane, can be used as an addition to concrete though a proper mix design (Baturkin et al. 2021; Revilla-Cuesta et al. 2023b).

Despite the robust appearance of concrete, thermal fluctuations when in service can significantly affect its service life (Ferronato et al. 2023). Thermal stress due to temperature fluctuations must always be computed at the design stage of any concrete component (EC-2, 2010; ACI 2014). Moreover, the presence of alternative materials within the concrete to improve its sustainability (J. Xu et al. 2022a, b; Çeçen et al. 2023; Li et al. 2023), such as recycled concrete, slag, and plastic aggregates, will always affect its response to temperature fluctuations. Recycled concrete aggregate exposed to cyclic thermal fluctuations causes micro-cracking within the interfacial transition zones that reduces strength (Revilla-Cuesta et al. 2023a). The high density of slag aggregate increases the capability of the concrete to withstand increasing temperatures with less loss of strength (Beaucour et al. 2020). Finally, the application of high temperatures to a concrete made with plastic aggregates causes a high loss of strength, due to the melting of plastic particles (Tariq et al. 2021).

RCWTB incorporates balsa wood and polyurethane particles in its composition (Revilla-Cuesta et al. 2023b). These particles act as aggregate in the concrete, partially replacing natural aggregate. However, both materials are sensitive to the application of high temperatures, at which point they may melt (polyurethane) or burned (balsa wood) (Tariq et al. 2021). Moreover, if these temperatures occur under conditions of high humidity, balsa wood can be deteriorated more pronouncedly (Wang et al. 2021). Given this situation, the behavior of concrete-containing additions of RCWTB when exposed to high temperatures and thermal fluctuations in a humid atmosphere is studied in this research. Thus, the aim is to evaluate the levels of deterioration of the performance of concrete-containing RCWTB under those environments. Clearly defining this behavior will allow determining the suitability of the concrete with this waste in applications where concrete is exposed to similar conditions, such as pavements, bridge decks, or cooling towers (Beaucour et al. 2020; Santamaría et al. 2020; Tamayo et al. 2022).

## Materials and methods

### Raw materials

**Fig. 2:**
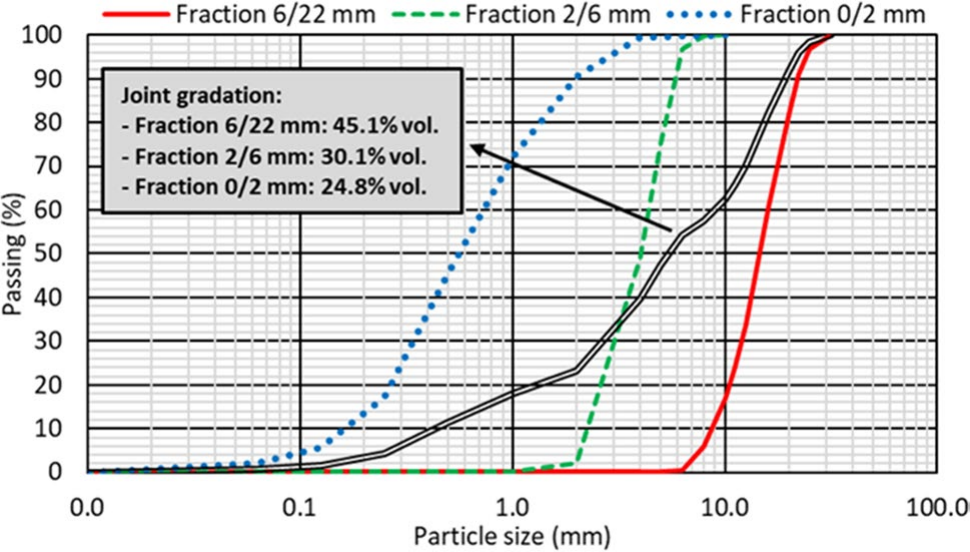
Individual and joint gradation of crushed siliceous aggregate fractions

The mixes for this study were prepared with conventional raw materials, as the purpose was to study the effects of additions of RCWTB. The concrete was therefore prepared with ordinary Portland cement CEM II/A-L 42.5 R, with a limestone addition of between 6 and 20% in weight according to EN 197–1 (EN-Euronorm) to improve sustainability; tap water; and two plasticizers (a polycarboxylate-based intended to guarantee suitable hydration of all the cement grains and a high-range water reducer) in order to maximize concrete strength while reaching proper workability for a suitable period of time (Shanahan et al. 2016); and three fractions of crushed siliceous aggregate sized 0/2 mm, 2/6 mm, and 6/22 mm. Their granulometry and the physical properties are shown in Fig. 2 and Table 1, respectively, in which their continuous gradations and conventional densities and water-absorption levels can be seen.

**Table 1:** Physical properties of crushed siliceous aggregate as per EN 1097–6 (EN-Euronorm)

	SSD density (kg/dm^3^)	Water absorption in 24 h (% wt.)
Fraction 6/22 mm	2.59	1.66
Fraction 2/6 mm	2.58	1.83
Fraction 0/2 mm	2.62	0.52

*SSD*, saturated surface dry

**Fig. 3:**
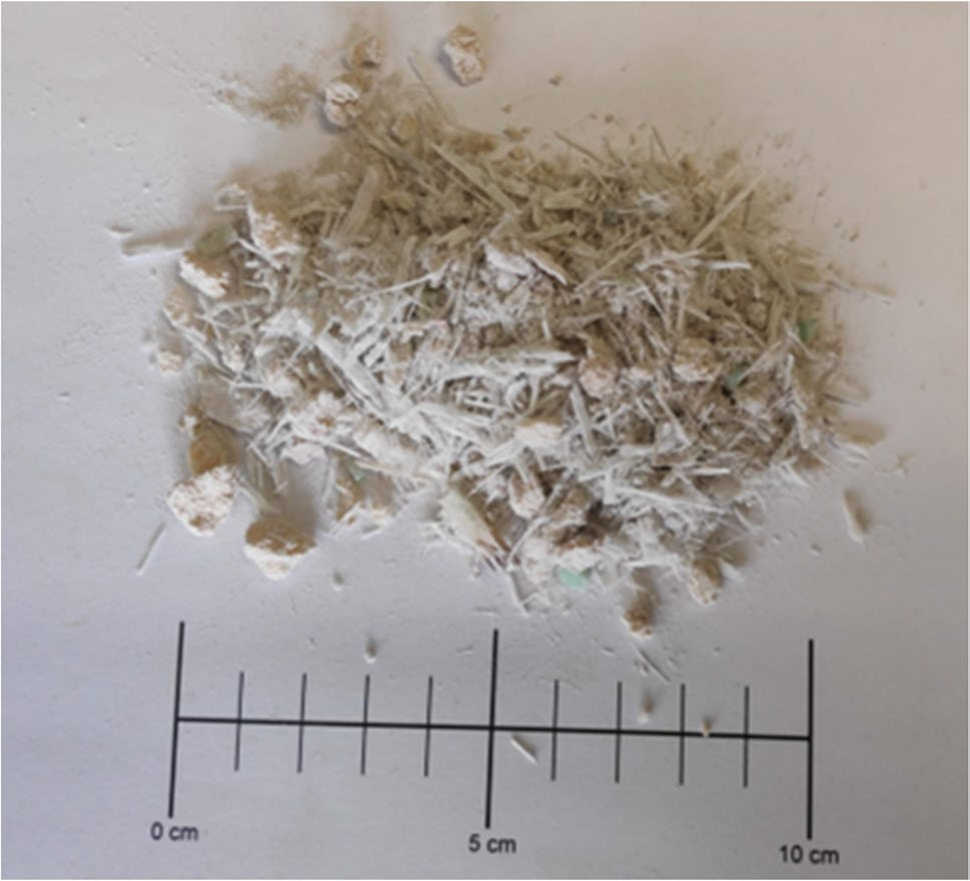
Raw-crushed wind-turbine blade

RCWTB (Fig. 3) was obtained by crushing sandwich panels similar to the one shown to the left of Fig. 1 using a knife mill designed for crushing plastic containers. Subsequently, it was passed through a 10-mm-aperture sieve. All the material that was retained in the sieve was once again crushed and sieved. The resulting RCWTB material was composed of GFRP-composite fibers (66.8% wt. of RCWTB; average length of 13.1 mm); approximately spherical balsa-wood particles (6.3% wt. of RCWTB; density of 0.33 kg/dm^3^); polyurethane particles (8.3% wt. of RCWTB); and a mixture of glass fibers disintegrated from the epoxy matrix and small particles of balsa wood and polyurethane that were not separable by mechanical sieving (18.6% wt. of RCWTB). RCWTB had a real density of 1.63 kg/dm^3^. More details of this material can be found in another paper (Revilla-Cuesta et al. 2023b). The RCWTB was used in this research without any previous surface treatment in order to analyze the suitability of the material composing wind-turbine blades in its original state (Tao et al. 2023).

### Mix design

First, the reference mix (0% RCWTB) was designed and produced. The design had two objectives: on the one hand, a slump between 10.0 and 15.0 cm (slump class S3, EN 206 as *per* (EN-Euronorm)); on the other hand, a minimum cube compressive strength of 45 MPa. A concrete mix that satisfies both requirements is generally suitable for all types of applications (EC-2, 2010; ACI 2014). For this purpose, the proportioning specifications of Eurocode 2 (EC-2, 2010) were followed in the design phase, adjusting the amount of each aggregate fraction by optimizing the fit of the joint gradation to the Fuller’s curve by least squares (Fig. 2). Furthermore, concrete mixes with varying contents of both water and plasticizers were prepared previously to the definitive ones until the slump target was reached, thus accurately defining the amounts of water and plasticizers to be added.

Next, the RCWTB mixes, with the overall additions of the waste, were designed. The balsa-wood and polyurethane particles and the GFRP-composite fibers served as aggregate and fibers, respectively. These additions also reduced the cement content per m^3^ of the concrete and its carbon footprint. Quantities of 1.5%, 3.0%, 4.5%, and 6.0% in volume of RCWTB were added, adjusting the content of water and plasticizer, so that the slump class remained constant. Thus, all the mixes were of class S3, guaranteeing the comparability of the results.

The five mixes prepared in that way were labeled with the letter *M* followed by the RCWTB percentage. So, for example, the *M3.0* mix incorporated 3.0% RCWTB. The mix compositions are shown in Table 2.

**Table 2:** Composition of concrete mixes (kg)

Mix		*M0.0*	*M1.5*	*M3.0*	*M4.5*	*M6.0*
Cement		320	320	320	320	320
Plasticizers	Plasticizer 1	2.20	2.62	3.04	3.46	3.88
	Plasticizer 2	1.10	1.31	1.52	1.73	1.94
Water		128	133	137	142	146
Crushed siliceous aggregate	Fraction 6/22 mm	900	900	900	900	900
	Fraction 2/6 mm	600	600	600	600	600
	Fraction 0/2 mm	500	500	500	500	500
RCWTB		0.0	24.5	49.0	73.5	98.0
Mix volume (m^3^) *		1005.3	1025.9	1045.5	1066.1	1085.8

^*^ Adding RCWTB without changing the content of the other components led to a decrease in the amount of cement per m^3^

### Production of concrete and specimens

A five-stage mixing process was implemented to ensure the highest possible level of concrete workability. The stages were intended to maximize aggregate water absorption during mixing (Güneyisi et al. 2014), to compensate for the expected loss of workability when RCWTB was added, due to the presence of GFRP-composite fibers (Ortega-López et al. 2022), and to ensure a uniform distribution of RCWTB in the concrete mass. These stages involved: (1) the addition of all the aggregates and 30% of the mix water shown in Table 2, and mixing during 3 min; (2) the addition of the cement together with the remaining mix water, and mixing during 3 min; (3) the addition of half of the admixtures dissolved in a quarter liter of water, and mixing during 2 min; (4) the addition of RCWTB, and mixing during 2 min; (5) the addition of the other half of the admixtures dissolved in another quarter liter of water, and concrete mixing for 5 min.

After mixing, the slump test as specified in EN 12350–2 (EN-Euronorm) was performed on a fresh-concrete sample and then 10 × 10 × 10-cm cubic specimens were produced for all the other tests. All the tests were performed on three specimens; the results presented in this paper showing the average and standard deviation of the three individual results. The specimens were demolded 24 h after concrete manufacture and stored in a standardized humid chamber at a temperature of 20 ± 2 °C and a humidity level of 90 ± 5% until an age of 90 days. The strength was considered to have stabilized at this age (Ortega-López et al. 2022) and, therefore, all tests were conducted when the concrete was 90-day old.

### Experimental procedure

#### Reference values

First, tests were performed to define the reference values of hardened density as per EN 12390–7 (EN-Euronorm) and compressive strength according to EN 12390–3 (EN-Euronorm) with which to compare the results obtained after the temperature-moisture-fluctuation tests. Cubic specimens that had not previously been subjected to any test were used for that purpose. Moreover, the effective porosity of the concrete mixes was evaluated through the capillary-water-absorption test according to UNE 83982 (2008). To do so, the moisture content of the specimens was controlled by exposing them to a temperature of 20 ± 2 °C and a relative humidity of 60 ± 5% in a laboratory environment over 24 h as per UNE 83966 (2008). Then, and after having removed the skin from the opposite face to concrete pouring, the specimens were subsequently immersed in a 2-mm-thick layer of water until saturation. The effective porosity of the concrete was determined as the quotient between the difference in weight of the concrete at the beginning of the test and before saturation (water volume) and the geometric volume of the concrete specimen.

#### Behavior under simultaneous changes in humidity and temperature

After suitable conditioning of the concrete specimens as per UNE 83966 (2008), the analyses of concrete behavior under changes in humidity and temperature were conducted through moist/dry and alternating-sign-temperature-shock tests:

- The moist/dry test was performed by adapting the instructions of ASTM D559 (ASTM-International). The cubic concrete specimens were subjected to 30 moist/dry cycles. The moist phase for each cycle lasted 16 h and consisted of immersing the specimens in water at an indoor temperature (20 ± 2 °C). The dry phase involved oven drying the specimens at a temperature of 70 ± 2 °C for 8 h. In this way, extreme environmental conditions of exposure to rain and solar radiation were simulated, which can be found, for instance, on pavements (Ortega-López et al. 2022).
- The alternating-sign-temperature-shock test was also performed by exposing the concrete to ambient cycles. In each cycle, the specimens were placed for 8 h in a freezer at a temperature of – 20 ± 1 °C and then immersed in water at a temperature of + 70 ± 2 °C for 16 h. The freezing conditions were defined according to the freeze/thaw test, standard UNE 12390–9 EX (2008), while the heating phase was defined according to the moist/dry test, standard ASTM D559 (ASTM-International), although it was performed in a moist environment, to analyze possible deterioration of the balsa-wood particles within the RCWTB (Hirschmüller et al. 2018). The test was intended to simulate extreme environmental conditions of rain, solar heating, and potential frost, as can occur on pavements and bridge decks, for example, Santamaría et al. (2020) and Revilla-Cuesta et al. (2023a). Two different durations, 10 and 20 cycles, were considered to evaluate the impact of the number of cycles in the strength performance of concrete.

The weight and ultrasonic pulse velocity (UPV) were recorded every five cycles in the moist/dry test and every cycle in the alternating-sign-temperature-shock test to evaluate the micro-structural damage. This damage was verified when the tests ended through scanning electron microscope (SEM) analyses performed on portions from within different concrete specimens. In addition, the length of the specimen edges (8 edge lengths per specimen) was measured before and after these tests to evaluate the dimensional variation of concrete under those environmental conditions (Ortega-López et al. 2022). Finally, hardened density and compressive strength were also determined at the end of these tests, to study the deterioration of those properties in relation to the reference values. Compressive strength was the mechanical property analyzed since it is considered the reference property by standards (EC-2, 2010; ACI 2014), and because the addition of RCWTB is usually more detrimental for it (Revilla-Cuesta et al. 2024), so the damage to the RCWTB components caused by the variations in humidity and temperature could be quite negative for it.

#### Behavior under high-temperature shock

The high-temperature-shock test consisted of exposing the concrete specimens to a temperature of 200 ± 5 °C for both 3 and 7 days. An oven was used to achieve this temperature in a sustained manner, which simulated the working conditions in concrete constructions where high temperatures are reached, such as cooling towers and nuclear-power plants, and even a possible fire (Beaucour et al. 2020; Tamayo et al. 2022). After the high-temperature-shock test, the compressive strength was evaluated for the purposes of a comparison with the reference values. This mechanical property was chosen due to the same reasons as for the moist/dry and alternating-sign-temperature-shock tests. UPV variations before and after the test were also analyzed.

#### Statistical and environmental analysis

An ANOVA with a 95% confidence level was conducted to evaluate the significance of the behavioral changes caused by the temperature and moisture fluctuations. In addition, the influence of the different factors on the compressive-strength variations of the RCWTB concrete was further investigated through a multiple-regression predictive analysis. Finally, an environmental analysis of the mixes was also performed. For this purpose, the carbon footprint per cubic meter and per unit of compressive strength of the different mixes after each test conducted was calculated.

## Results and discussion

### Reference properties

**Fig. 4:**
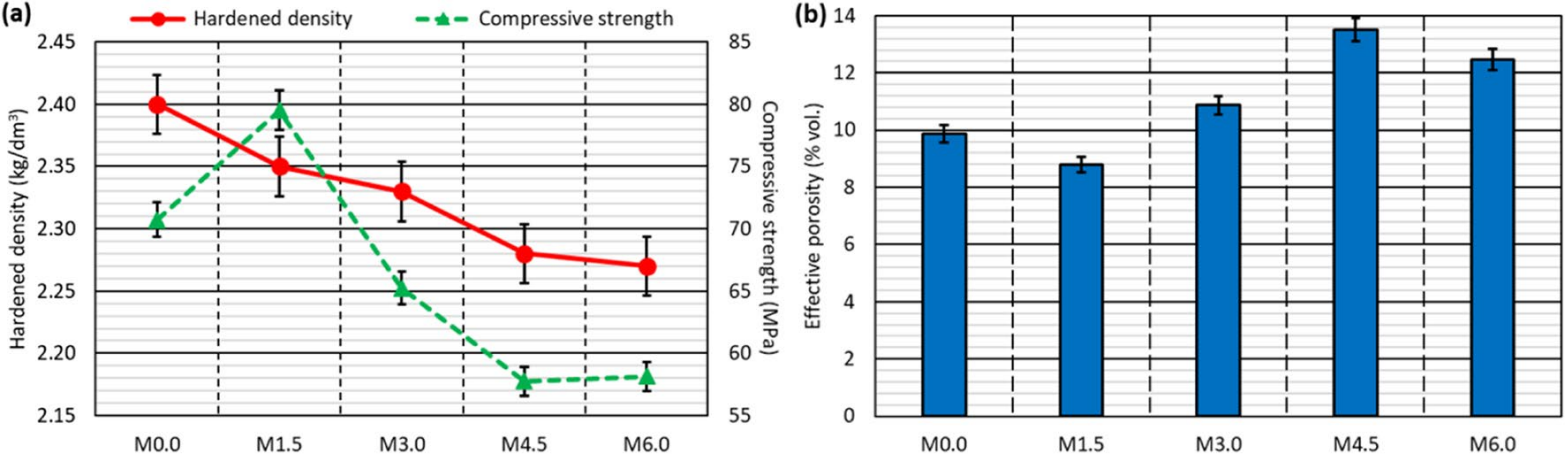
Reference 90-day hardened properties of concrete mixes: **a** density and cubic-specimen compressive strength; **b** effective porosity

The reference values of hardened density and compressive strength, measured on cubic concrete specimens not previously subjected to moisture and temperature variations, are shown in Fig. 4. The effective-porosity values are also depicted in the same figure.

As can be seen in Fig. 4a, the hardened density decreased in an approximately linear manner as the RCWTB content increased, falling from 2.40 kg/dm^3^ in the *M0.0* reference mix to 2.27 kg/dm^3^ in the *M6.0* mix. This decrease was due to the lower density of RCWTB compared to aggregate and cement (Baturkin et al. 2021), and to the increased content of both water and plasticizers, both even less dense than the RCWTB, as the amount of that waste material increased (Andreu and Miren 2014). The increase in concrete porosity when adding RCWTB also contributed to lowering the hardened density (Ouyang et al. 2020).

From a general perspective, the compressive strength (Fig. 4a) decreased as the RCWTB content increased. However, the lowest recorded strength was 57.8 MPa, which points to the quality of the concrete strength, despite the addition of large amounts of RCWTB (EC-2, 2010; ACI 2014). Two mixes disrupted this general trend: on the one hand, the *M1.5* mix, with a 12.4% higher strength than that of the *M0.0* mix, possibly due to the positive effect of the GFRP-composite fibers in terms of cementitious-matrix bridging (G. T. Xu et al. 2022a, b); on the other hand, the *M6.0* mix, almost identical to the *M4.5* mix in terms of strength, which highlights the bridging effect of the GFRP-composite fibers when adding high amounts of RCWTB that counteracted the negative effect of the particles of balsa wood and polyurethane.

The trend exhibited by the effective porosity (Fig. 4b), increasing with the RCWTB from a general approach, was closely linked to that of the compressive strength. Decreased porosity therefore implied an increase in strength, while a reduction in strength occurred in the more porous mixes, as was also found in other concrete types (Revilla-Cuesta et al. 2021). It is important to emphasize that the effective porosity of those mixes not only reflected the percentile volume of accessible pores in the cementitious matrix but might also be partially due to the volume of balsa-wood particles that can absorb water due to their high porosity (Jang and Kang 2022).

### Moist/dry test

The moist/dry test is a relevant durability test for construction materials (Ortega-López et al. 2018). As described in the “Experimental procedure” section, this test consisted of 30 cycles alternating water immersion at 20 ± 2 °C with oven drying at 70 ± 2 °C by adapting the ASTM D559 standard (ASTM-International). It involved simulating environmental conditions of humid and rainy climates (water-immersion phase), alternating with high temperatures (oven-drying phase) (Ortega-López et al. 2022). Water absorption and thermal shock due to sudden oven drying can affect a cement-based material internally, causing micro-cracking, due to the different thermal deformability of the materials that compose it (Revilla-Cuesta et al. 2023a). The recurrent presence of excessive humidity and its rapid decrease during oven drying can also damage the interfacial transition zones (Sun et al. 2022). Finally, it should be noted that the moist/dry test is a laboratory test which takes the environmental conditions described to an extreme situation, i.e., humidity conditions (water immersion) and (oven) drying at 70 °C, which merely simulate real climatic conditions. Nevertheless, the test results offer a useful approximation of how cement-based materials may behave under such conditions (Ortega-López et al. 2022).

**Fig. 5:**
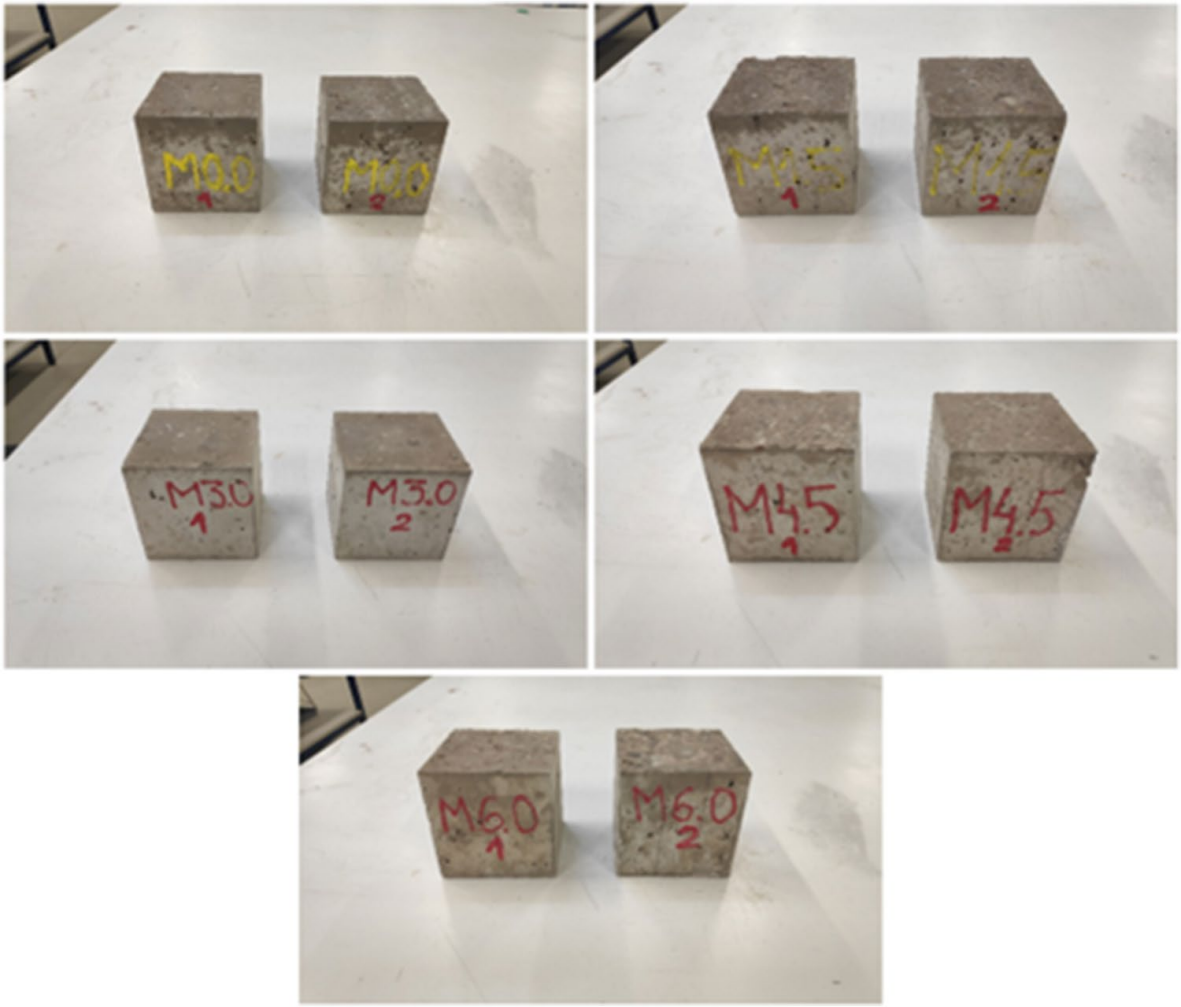
External appearance of two representative specimens *per* concrete mix after the moist/dry test

**Fig. 6:**
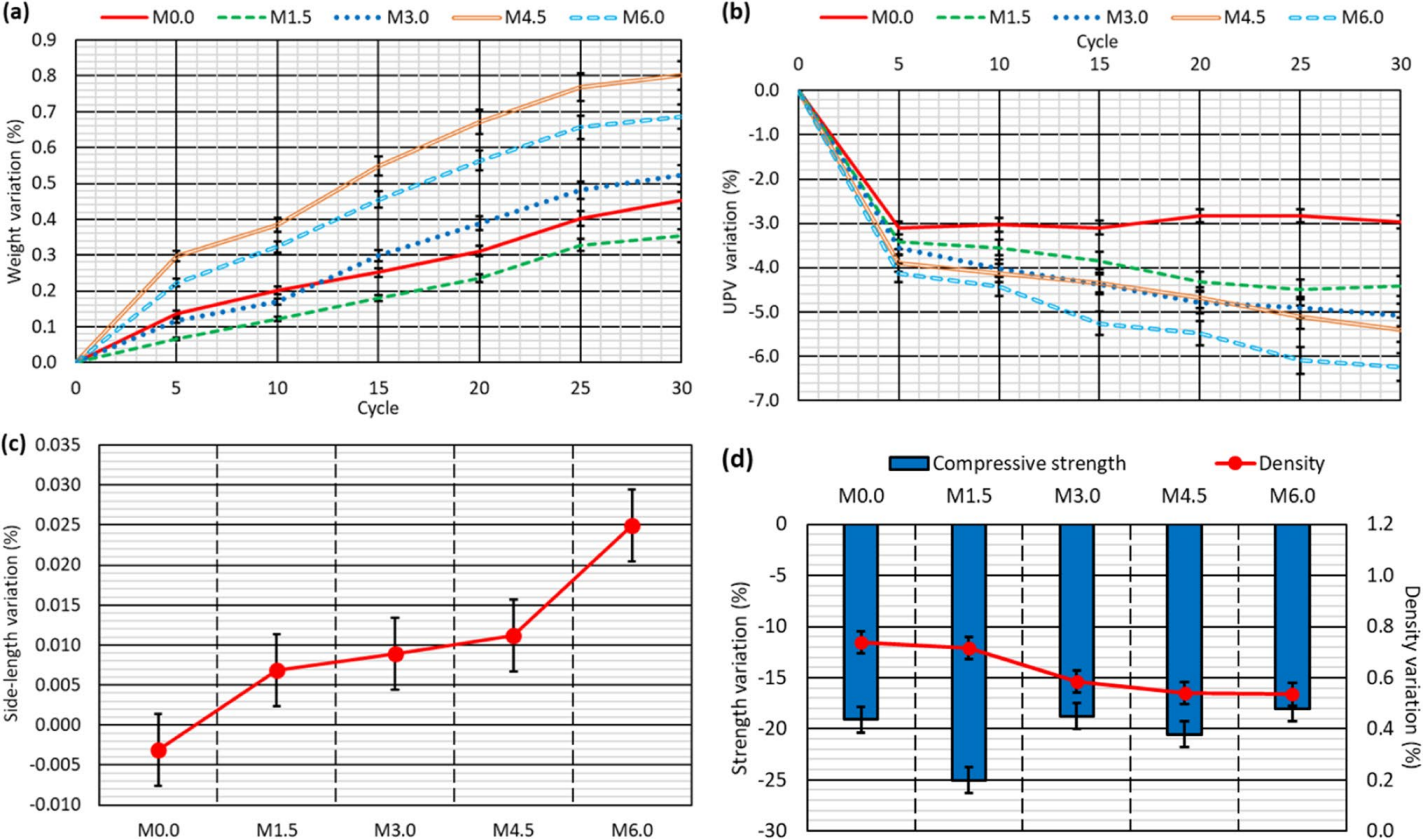
Results of the moist/dry test: **a** weight evolution; **b** UPV variation; **c** dimensional stability; **d** variation of compressive strength and density

**Fig. 7:**
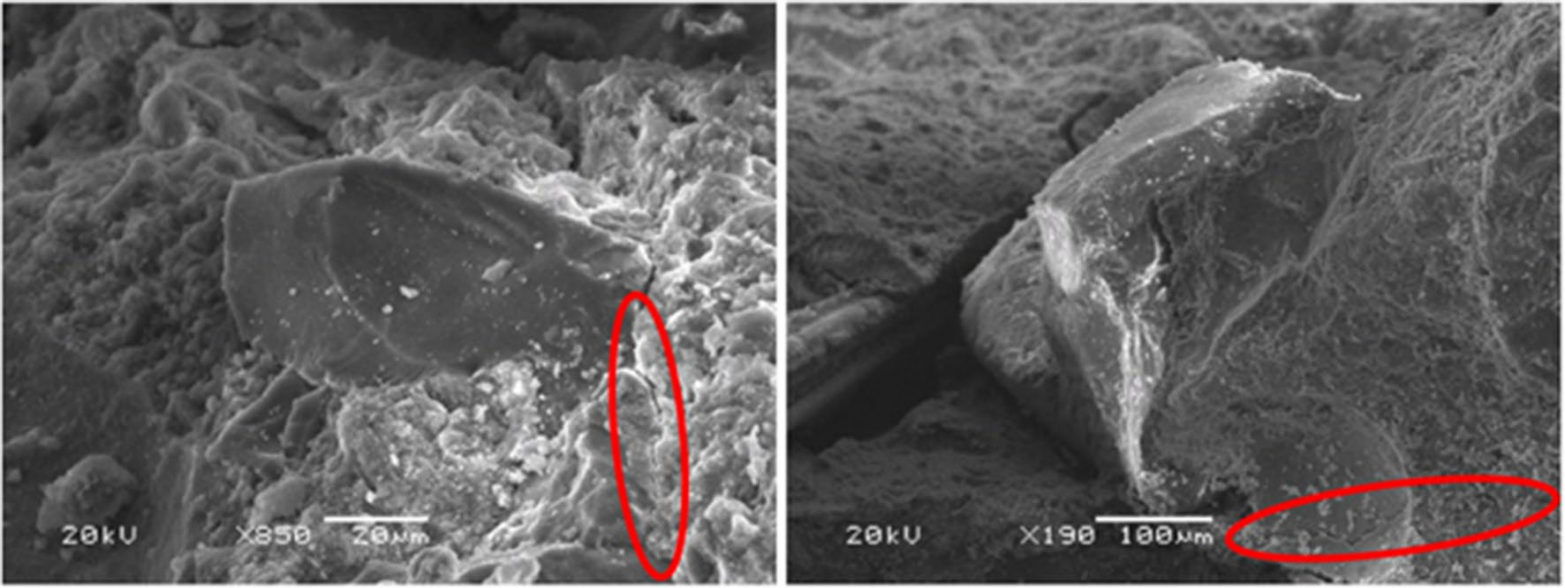
SEM analysis after the moist/dry test (micro-cracks encircled in red): *M4.5* mix (left) and *M6.0* mix (right)

Cubic specimens of the five concrete mixes were subjected to a moist/dry test as described in the “Experimental procedure” section. At the end of the test, the concrete specimens showed no visible signs of damage, as shown in Fig. 5, except for some dark colored marks due perhaps to deterioration of the specimen skin (Santamaría et al. 2020) and a slight rounding of the corners. However, the computation of the specimen weight and the UPV readings every five cycles pointed to progressive damage to the concrete throughout the test:

- On the one hand, the concrete specimens progressively increased in weight, due to their increased water absorption throughout the test (Fig. 6a). Thus, water-absorption levels were between 0.05 and 0.30% wt. after five cycles and between 0.25 and 0.80% wt. at the end of the test. This continuous increase in the amount of absorbed water may be attributed to the appearance of micro-cracking within the cementitious matrix, due to temperature changes and the different thermal deformability of the concrete components (Revilla-Cuesta et al. 2022), which was favored by the presence of GFRP-composite fibers in the RCWTB that disrupted the continuity of the cementitious matrix (Ortega-López et al. 2022). The higher or lower initial water absorption of the mixes was in accordance with the effective-porosity values (Fig. 4b) (Hamada et al. 2023), which were not linearly proportional to the RCWTB content. The most porous mixes also experienced the highest increase in water-absorption levels throughout the test, due perhaps to their larger pore volume, which may have favored micro-cracking (Revilla-Cuesta et al. 2023a).
- On the other hand, the UPV readings showed a continuous decrease throughout the whole test (Fig. 6b and Table 3). Thus, the UPV reading for the *M6.0* mix was 6.0% lower at the end the test. A result that underlines the continuous deterioration within the concrete following the application of moist/dry cycles (Ortega-López et al. 2022) was also noted for the evolution of sample weight. However, unlike water absorption, the UPV readings were lower as the RCWTB content increased, so rather than dependent on porosity. Micro-cracking of the cementitious matrix was found in this test, although the RCWTB components, all sensitive to high temperatures, might also have been negatively affected (Rani et al. 2021; Tariq et al. 2021; Jang and Kang 2022).
- SEM analyses performed after completion of the test (Fig. 7) on concrete fragments of the *M4.5* and *M6.0* mixes, the ones with the highest damage, verified the existence of micro-cracking that appeared primarily in the interfacial transition zones. Furthermore, it seemed that these micro-cracks were subsequently easily propagated through the pores. This reinforced the conclusions on internal damage derived from the analysis of weight and UPV evolutions throughout the moist/dry test.

**Table 3:** UPV, compressive-strength, and density measurements (average values) of the specimens tested to moist/dry

		*M0.0*	*M1.5*	*M3.0*	*M4.5*	*M6.0*
Before the test	UPV (km/s)	4.036	3.922	3.937	3.854	3.711
After the test	UPV (km/s)	3.917	3.749	3.737	3.646	3.479
	Compressive strength (MPa)	57.2	59.6	53.0	45.9	47.7
	Density (kg/dm^3^)	2.42	2.37	2.34	2.29	2.28

The temperature fluctuations to which the concrete was exposed caused its dimensions to alter, due either to expansion or to contraction (Smith and Tighe 2009). In addition, the repeated application of these thermal changes can cause remaining strain to appear at the end of the test (Revilla-Cuesta et al. 2022), as shown in Fig. 6c. On the one hand, the *M0.0* mix specimens (0% RCWTB) shortened by 0.003%, which shows that the micro-cracking experienced by the reference mix was very limited and in no way affected its dimensional stability (Santamaría et al. 2020). However, remaining strain appeared in all the mixes with RCWTB that increased with the content of this waste, reaching length increases of 0.025% in the *M6.0* mix. The application of high temperatures may have permanently increased the volume of the polyurethane and balsa-wood particles (Tariq et al. 2021), and the latter may also have been affected by the notable moisture changes during this test (Wang et al. 2021). In addition, the GFRP-composite fibers could have been permanently increased in length by thermal expansion of the epoxy resin (Rani et al. 2021). These aspects could have led to the emergence of a remaining strain within the concrete.

At the end of the moist/dry test, the density and compressive strength of the mixes were measured (Table 3) and compared with the reference values (Fig. 4a). A comparison is depicted in Fig. 6d.

- Density levels increased due to the increased amount of water within the concrete after the test that resulted in weight gain (Fig. 6a). On the contrary, density decreased due to the larger specimen dimensions after the test (Fig. 6c). Combining both aspects resulted in increased density (Fig. 6d), as the increase in weight was more relevant. However, any increase in density was minimal (less than 1% in all cases), which reflects the previously discussed question of micro-cracking (Ortega-López et al. 2018), evidence of which is the increased water absorption levels of the concrete (Santamaría et al. 2018). No clear trend was observed following the addition of RCWTB.
- Compressive strength decreased by 15% to 25% after the moist/dry test (Fig. 6d). Clearly, the evident micro-cracking of the concrete revealed by all the non-destructive properties under evaluation resulted in a strength decrease. However, this decrease showed no clear trends with regard to either the RCWTB content or the porosity of the mixes. In fact, the *M1.5* mix suffered the greatest loss of compressive strength (− 25%) despite being the least porous and having a very low RCWTB content. It is thought that the GFRP-composite fibers contained in the RCWTB, although they may have slightly deteriorated due to epoxy-resin damage after exposure to high temperatures (Rani et al. 2021), exercised a bridging effect within the cementitious matrix (Baturkin et al. 2022), meaning that the negative effects on concrete strength of micro-cracking and damage to the particles of balsa wood and polyurethane were less noticeable (Ortega-López et al. 2022). Thus, the GFRP-composite fibers compensated for the negative effect of balsa wood and polyurethane particles under moist/dry cycling, leading to a strength behavior similar to that of conventional concrete without RCWTB.

### Alternating-sign-temperature-shock test

The alternating-sign-temperature-shock test was intended to expose the concrete to more extreme environmental conditions than in the moist/dry test. For this purpose, cubic concrete specimens were subjected to either 10 or to 20 cycles of two phases, under wet and dry (freezing) environments, respectively, as described in the “Experimental procedure” section. The wet environment involved immersion of the specimens in water at + 70 ± 2 °C, thereby combining the effects of exposure to high temperatures and moisture described for the moist/dry test (Revilla-Cuesta et al. 2022; Sun et al. 2022). The dry environment was obtained by placing the specimens in a freezer at – 20 ± 1 °C, so that the water contained inside the concrete froze and increased in volume, which usually favors the internal micro-cracking of concrete (Güneyisi et al. 2014). In that way, environmental conditions corresponding to warm and rainy climates and harsh frosts were simultaneously simulated (Ortega-López et al. 2018). In short, very extreme climatic conditions were simulated through a single laboratory test to validate the concrete-containing RCWTB under a wide range of climatic conditions (Ortega-López et al. 2022).

**Fig. 8:**
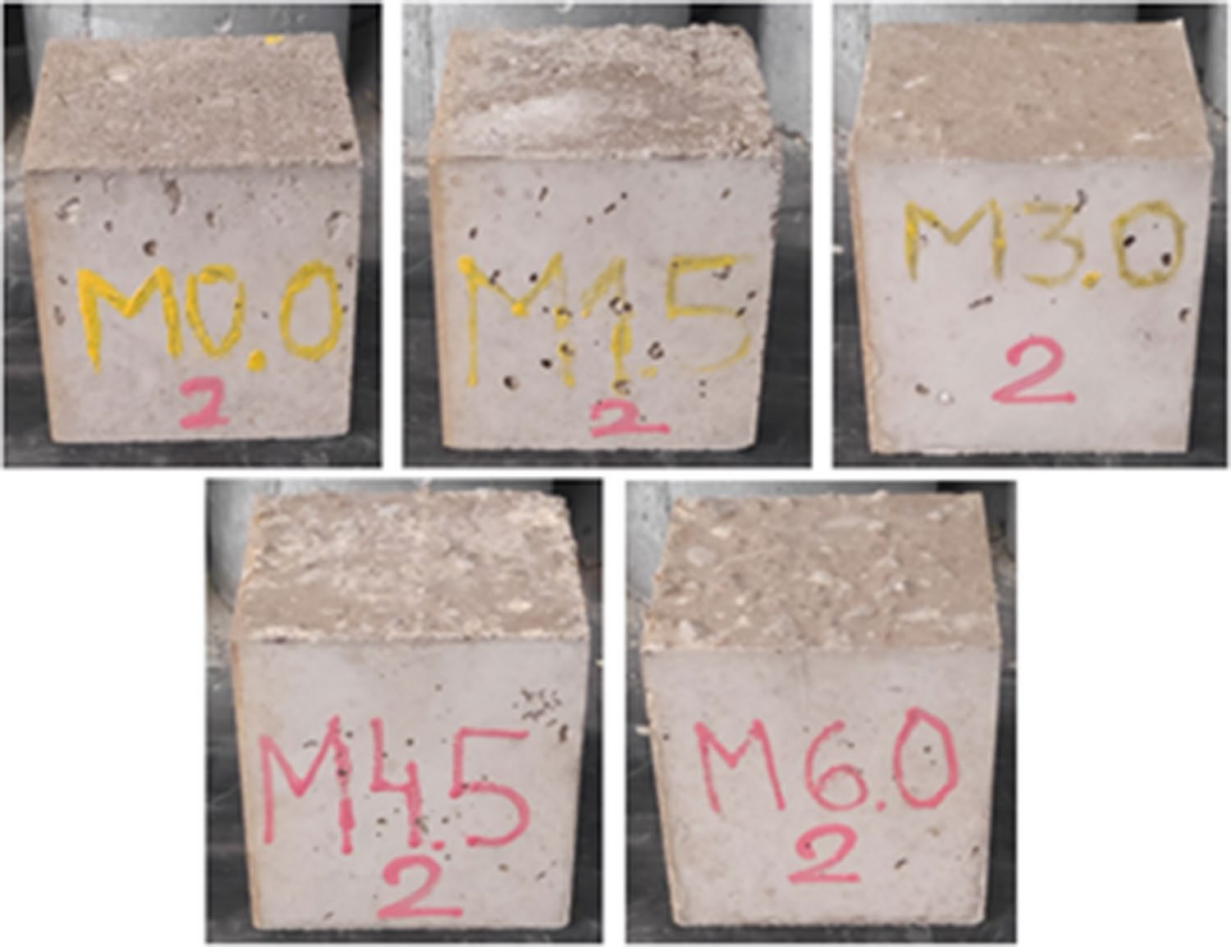
External appearance of a representative specimen after the 20-cycle alternating-sign-temperature-shock test

**Fig. 9:**
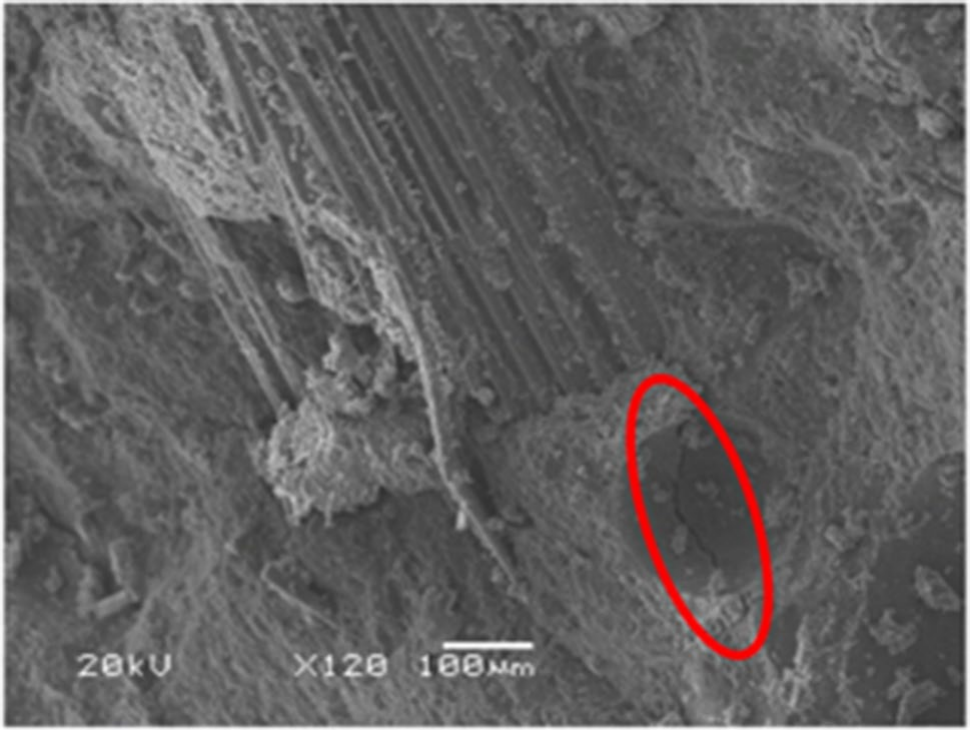
SEM image of the *M4.5* mix after the 20-cycle alternating-sign-temperature-shock test (micro-cracks encircled in red)

**Fig. 10:**
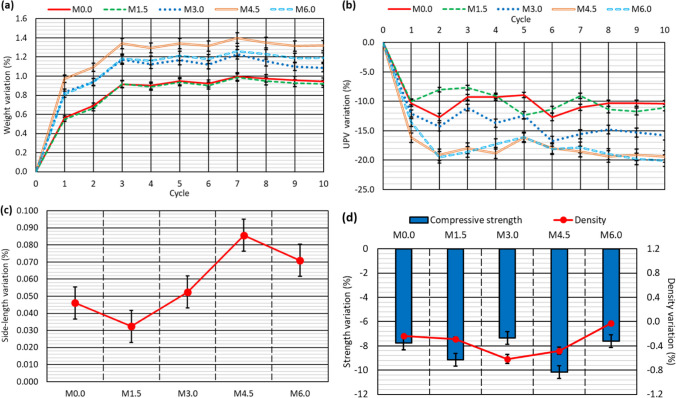
Results of the alternating-sign-temperature-shock test with a duration of 10 cycles: **a** weight evolution; **b** UPV variation; **c** dimensional stability; **d** variation of compressive strength and density

**Fig. 11:**
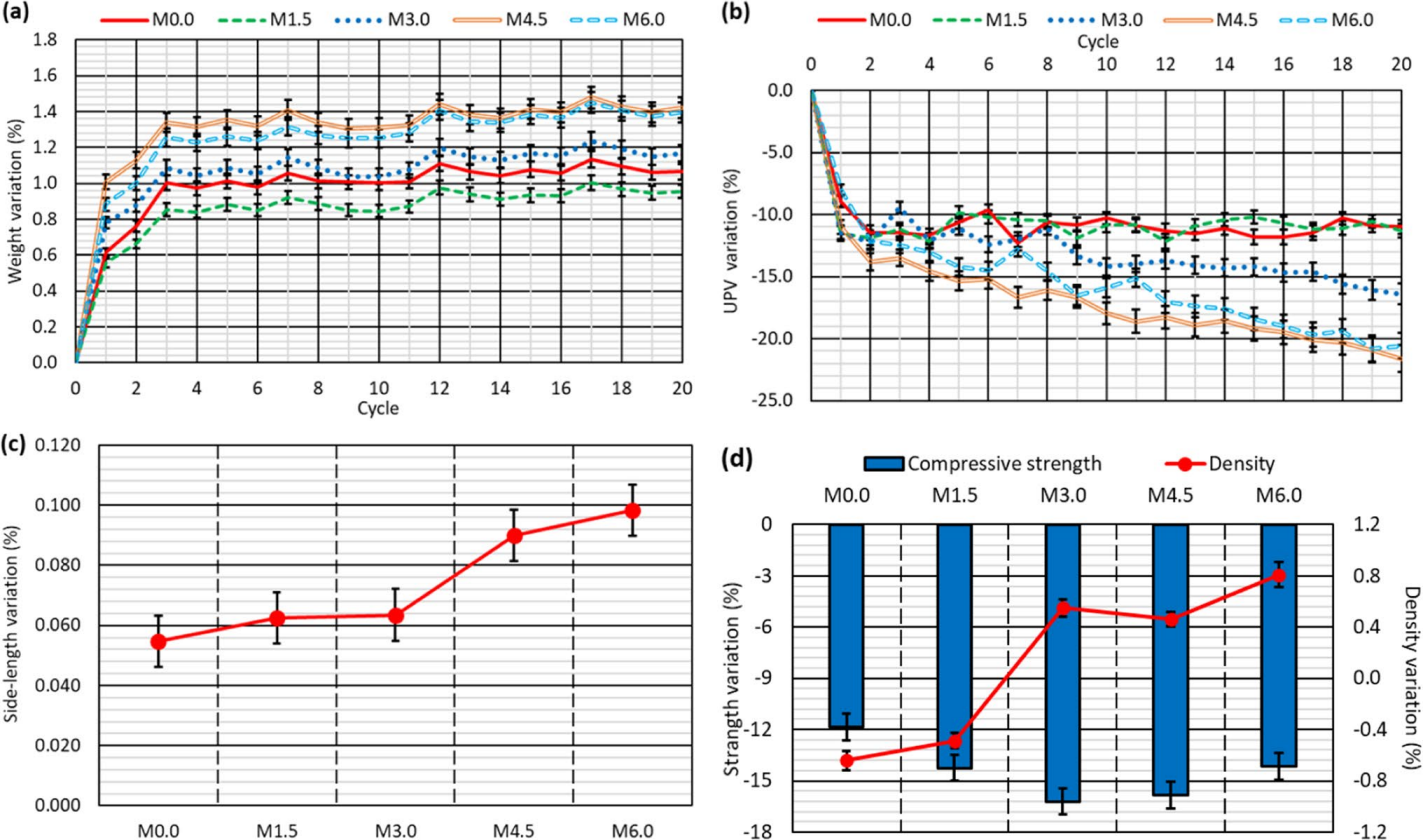
Results of the alternating-sign-temperature-shock test with a duration of 20 cycles: **a** weight evolution; **b** UPV variation; **c** dimensional stability; **d** variation of compressive strength and density

At the end of the test, the specimens showed no external signs of deterioration (Fig. 8), but did internal damage (Fig. 9). The increased weight of the specimens (Figs. 10a and 11a) and their lower UPV readings (Table 4 and Figs. 10b and 11b) throughout the test did show the appearance of that internal damage in the concrete (Jones 1963), similar to what was found in the moist/dry test. The micro-cracking that appeared followed the same pattern as in the moist/dry test, although in this case cracking was also detected in some areas of the bond between the cementitious matrix and the GFRP-composite fibers. This phenomenon can be seen in Fig. 9, an image obtained through SEM analysis of a fragment of the *M4.5* mix after the 20-cycle alternating-sign-temperature-shock test, and may be due to the application of negative temperatures (Revilla-Cuesta et al. 2023a).

**Table 4:** UPV, compressive-strength and density measurements (average values) on the specimens tested to alternating-sign-temperature shock

			*M0.0*	*M1.5*	*M3.0*	*M4.5*	*M6.0*
Duration of 10 cycles	Before the test	UPV (km/s)	4.054	3.978	3.895	3.877	3.732
	After the test	UPV (km/s)	3.632	3.536	3.280	3.125	2.982
		Compressive strength (MPa)	65.2	72.2	60.5	51.9	53.7
		Density (kg/dm^3^)	2.41	2.36	2.33	2.28	2.28
Duration of 20 cycles	Before the test	UPV (km/s)	4.123	4.047	3.935	3.899	3.794
	After 10 cycles	UPV (km/s)	3.698	3.606	3.376	3.197	3.191
	After the test	UPV (km/s)	3.669	3.590	3.290	3.057	3.012
		Compressive strength (MPa)	62.3	68.2	54.7	48.6	49.9
		Density (kg/dm^3^)	2.40	2.36	2.36	2.30	2.30

The variations of weight and UPV throughout the alternating-sign-temperature-shock test occurred more abruptly than in the moist/dry test. Thus, the main variation of both magnitudes took place in the first 2–3 cycles, increasing very slightly in the rest of the test. It appears that the micro-cracking damage and the deterioration of the RCWTB components caused by the freezing phase were concentrated within the initial exposure time of the concrete to these conditions, as was also found with regard to other concretes produced with alternative raw materials (Güneyisi et al. 2014; Fiol et al. 2020). The increased damage that occurred during the rest of the test is thought to have been due to the thermal shock (Ortega-López et al. 2018), identical to what was encountered in the moist/dry test, but it was much less noticeable than the damage during the first cycles. Thus, the extensive damage to the concrete at the start of the test hardly differed after the concrete had been when exposed to either 10 cycles (Fig. 10a and b) or to 20 cycles (Fig. 11a and b). The differences in weight gains and the lower UPV readings between both numbers of the cycles was 0.10–0.15% and 1.50–2.00% in absolute values, respectively. Finally, as in the moist/dry test, the micro-cracking that propagated due to mix porosity (Fig. 4b) appeared to have controlled the weight increase due to water absorption (Revilla-Cuesta et al. 2023a), while the lower UPV readings were attributable to the deterioration of the RCWTB components with cycled aging (Rani et al. 2021; Tariq et al. 2021; Jang and Kang 2022). The variations of both magnitudes were greater than in the moist/dry test, although the difference was more notable regarding UPV (approximately four times higher), which might point to further deterioration of the polyurethane and balsa-wood particles, and the GFRP-composite fibers, under freezing conditions.

The specimens underwent remaining strain resulting from the internal damage described above (Ortega-López et al. 2022), which was detected by measuring their side lengths. The percentile variations of side length after the alternating-sign-temperature-shock test are shown in Figs. 10c and 11c for the tests with durations of 10 and 20 cycles, respectively. Several aspects can be highlighted in relation to the dimensional stability of the mixes during this test:

- First, the openings of the micro-cracks that occurred in the cementitious matrix as a consequence of the application of sub-zero temperatures appear to have been greater than the openings that occurred as a consequence of the thermal shock in the moist/dry test (Revilla-Cuesta et al. 2022). It could also be linked to the lower UPV readings during this test (Figs. 10b and 11b) (Jones 1963). All the concrete mixes therefore experienced permanent expansion. In fact, the specimens of the *M0.0* reference mix, whose dimensional stability was not affected in the moist/dry test, showed an increase in their side length of 0.046% after 10 cycles and 0.055% after 20 cycles.
- Second, the higher the RCWTB content, the greater the increase in side length, which could be due to the higher micro-cracking in these mixes and the deterioration that the RCWTB components underwent during freezing (Rani et al. 2021; Wang et al. 2021). Thus, the side-length increase of the *M6.0* mix was 0.098% after 20 cycles, 0.40% more in absolute terms than the reference mix. However, it is also true that the higher the number of cycles, the more pronounced the trend, due to the greater damage to the concrete (Santamaría et al. 2018).
- The higher the number of cycles, the greater the remaining strain of the specimens, but the difference was small. In absolute terms, the increase in the side length after 20 cycles was 0.10% higher than after 10 cycles. It once again shows that the main damage to the concrete occurred during the initial cycles when exposed to freezing conditions (Ortega-López et al. 2018).

The density and compressive strength of the mixes at the end of the test were used to verify the internal damage to the mixes (Table 4). Both properties varied in comparison with the reference values (Fig. 4a), as shown by the alternating-sign-temperature-shock test results, depicted in Figs. 10d and 11d, for 10 and 20 cycles, respectively.

- The concrete mixes underwent greater expansion in this test than in the moist/dry test. It meant that, despite the increase in weight due to water absorption, the density of all the mixes decreased after 10 cycles, due to the increased volume of the specimens (Santamaría et al. 2020). Internal damage to the specimens following micro-cracking due to thermal shock was greater after 20 cycles (Tariq et al. 2021), which caused the mixes with contents higher than 3.0% RCWTB to present an increase in density, due to their higher levels of water absorption (Ortega-López et al. 2022), with a trend similar to that observed in the moist/dry test. In all cases the variations were small, in the order of 1.0%, although the increase in RCWTB content led to a greater increase in density after the application of 20 cycles, due perhaps to the damage to the RCWTB components during the test (Fonte and Xydis 2021; Rani et al. 2021).
- Internal damage to the concrete during the test resulted in a reduction in compressive strength. This behavior was observed in all the mixes, including the *M0.0* reference mix, but increased with the additions of RCWTB. It was due to the fact that RCWTB favored internal damage, because the GFRP-composite fibers interrupted the continuity of the cementitious matrix (G. T. Xu et al. 2022a, b), promoting micro-cracking, and the polyurethane and balsa-wood particles were damaged during the test (Jang and Kang 2022). The negative effect of RCWTB was mainly observed when increasing the number of cycles, as no clear trend of the effect of RCWTB was detected after the application of 10 cycles (Fig. 10d), but the loss of compressive strength increased to 3.0% RCWTB and then stabilized after the application of 20 cycles (Fig. 11d). The increased damage with cycling is thought to be due to increased deterioration of the GFRP-composite fibers, which stitched the cementitious matrix less effectively as the number of cycles increased (Rani et al. 2021; Ortega-López et al. 2022). The lower level of damage to the GFRP-composite fibers with fewer cycles may have also caused the decrease in compressive strength to be greater after the moist/dry test (Fig. 6d) (Jones 1963), in which 30 cycles were conducted.

### High-temperature-shock test

The high-temperature-shock test was the last test, in which the cubic concrete specimens were exposed to temperatures of 200 ± 5 °C, over 3 and 7 days. The test conditions were defined according to the available laboratory equipment. The objective was to evaluate the deterioration of the balsa-wood and polyurethane particles contained in the RCWTB because of high temperatures (Tariq et al. 2021; Jang and Kang 2022). In addition, the GFRP-composite fibers might have also been affected during the test, due to the thermal sensitivity of both the epoxy resin and the glass of their composition (Rani et al. 2021), although there was less expected damage, due to their lower individual volume (Alshahrani et al. 2023). Finally, it should not be forgotten that partial decomposition of ettringite also occurs at these temperatures, which weakens the cementitious matrix (Revilla-Cuesta et al. 2022).

**Fig. 12:**
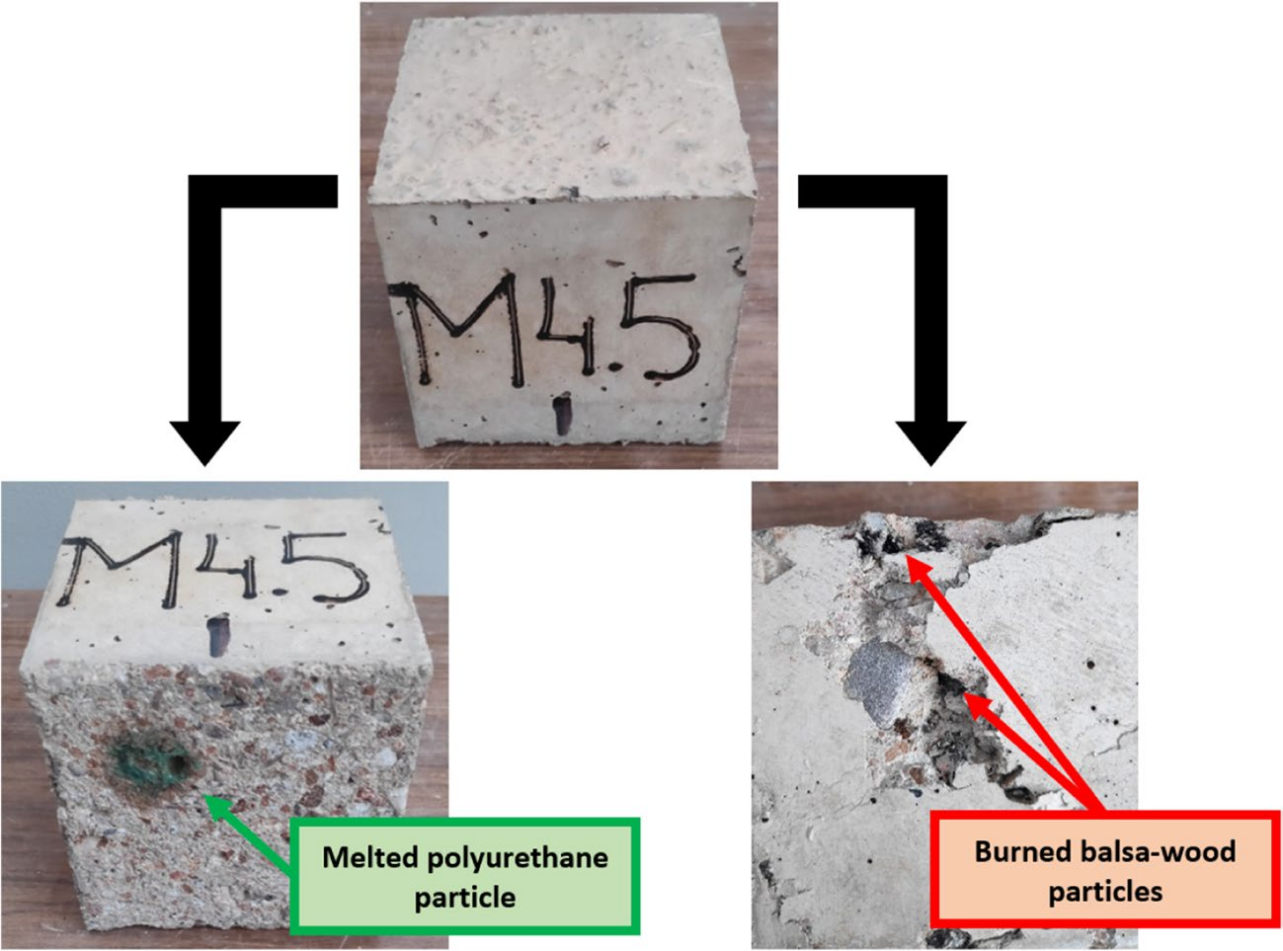
Visual analysis of the effects of the high-temperature-shock test on the components of RCWTB

The first aspect to be discussed is the visual analysis of the specimens conducted after the 7-day high-temperature-shock test. For this purpose, the skin was removed from the underside of some specimens before testing them under compression. In addition, the specimens were visually evaluated after breakage. No alteration of the characteristics of the GFRP-composite fibers was visible to the naked eye, but the other two RCWTB components were clearly affected, as shown in Fig. 12. Firstly, the polyurethane particles located in the outermost area of the specimens melted, leading to an increase in the macro-porosity of the concrete specimens (Ozturk et al. 2023). Then, it was observed that the high temperatures had burned the initially brown-colored balsa-wood particles (Fig. 3) that had turned black. Together with the expected decomposition of ettringite, the test conditions might have weakened the adhesion between the balsa-wood particles and the cementitious matrix within the interfacial transition zones (Revilla-Cuesta et al. 2023a). A reduction in concrete strength was therefore linked to both the melting of the polyurethane particles and the burning of the balsa-wood particles.

**Fig. 13:**
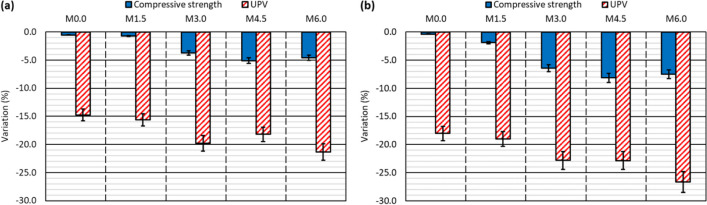
Variation of UPV and compressive strength after the high-temperature-shock test: **a** 3-day duration; **b** 7-day duration

Regarding the concrete properties measured during the high-temperature-shock test, UPV was recorded before the test, and UPV and compressive strength were measured after the test. All the results are detailed in Table 5. The compressive strengths were compared with the reference values (Fig. 4a), and the UPV values before and after the test were also compared. The variations of both properties throughout the test are depicted in Fig. 13. The main points arising from the analysis of these results can be grouped under three points:

- Firstly, in view of the test results, both UPV and compressive strength decreased during the high-temperature-shock test. The decrease in UPV was due to the increase in the porosity of the mix and the weakening of the interfacial transition zones (Jones 1963), which in turn reduced the compressive strength by weakening the concrete as a whole (Revilla-Cuesta et al. 2022). However, it should be noted that the *M0.0* mix, with 0% RCWTB, also experienced a slight decrease in UPV and strength, which proves the aforementioned decomposition of ettringite (Ortega-López et al. 2018).
- Secondly, the decrease in these properties was greater after exposure to high temperatures for 7 days. Overall, the maximum decreases after 3 days were around 20% for UPV and 5% for compressive strength, while after 7 days they were around 25% and 8%, respectively. Logically, longer exposure times aggravated both forms of deterioration (Beaucour et al. 2020), further worsening concrete performance. It should also be noted that the compressive-strength losses were very similar to those obtained after the 10-cycle alternating-sign-temperature-shock test, showing that both environmental conditions caused the same level of damage to concrete after similar exposure times.
- Finally, the effect of RCWTB can be analyzed. The increase in the added amount of this recycled waste material increased the concrete damage more clearly than in the other tests. Thus, the decrease in compressive strength for the *M0.0* mix after exposure over 7 days was 0.4%, while it reached 8.1% and 7.5% for the *M4.5* and *M6.0* mixes, respectively. However, it should also be noted that, regardless of the test duration, the decrease in strength was always slightly greater in the *M4.5* mix than in the *M6.0* mix (difference of 0.5–0.6% in absolute terms). A difference could be due to the beneficial bridging effect of the GFRP-composite fibers within the cementitious matrix (G. T. Xu et al. 2022a, b), although the higher porosity of the *M4.5* mix might have also influenced the result (Fig. 4b) (Pławecka et al. 2021). This particularity between these mixes was not evident in the UPV readings, which were conditioned by the damage caused to the polyurethane and balsa-wood particles, especially after the 7-day duration test, as was also found in another study (Santamaría et al. 2018). Thus, the lower UPV readings were linked to higher RCWTB contents.

**Table 5:** UPV and compressive-strength measurements (average values) on the specimens tested to high-temperature shock

			*M0.0*	*M1.5*	*M3.0*	*M4.5*	*M6.0*
3-day duration	Before the test	UPV (km/s)	4.025	3.948	3.892	3.806	3.753
	After the test	UPV (km/s)	3.431	3.331	3.121	3.113	2.954
		Compressive strength (MPa)	70.4	79.0	62.9	55.0	55.6
7-day duration	Before the test	UPV (km/s)	4.064	3.973	3.937	3.848	3.754
	After the test	UPV (km/s)	3.331	3.219	3.039	2.970	2.753
		Compressive strength (MPa)	70.5	78.0	61.3	53.4	54.1

### Statistical analysis

#### Analysis of variance (ANOVA)

The analyses performed so far in this study have revealed the effect of each factor on the behavior of concrete. However, it is necessary to evaluate whether these behavioral changes were sufficiently large to be significant (Ma et al. 2023). An ANOVA was conducted at a confidence level of 95% by considering all the individual results in the concrete specimens, to analyze the effects of both factors (RCWTB content and test duration) on concrete performance and their significance. The moist/dry test was analyzed with a one-way ANOVA, while a two-way ANOVA was implemented for the other two tests to consider the interaction between the RCWTB content and the test duration (Feng et al. 2022). Both the *p*-value and the homogeneous groups for each property of each test are detailed in Table 6.

**Table 6:** ANOVA (*α* = 0.05) of the variations caused by the temperature tests

Test	Property	*p*-value	Homogeneous groups
Moist/dry test (factor: RCWTB content)	Weight	0.0000	-
	UPV	0.0000	-
	Length	0.0874	*M0.0*, *M1.5*, *M3.0*, *M4.5,* and *M6.0*
	Compressive strength	0.0000	*M0.0* and *M3.0*
	Density	0.0001	*M0.0* and *M1.5*; *M4.5* and *M6.0*
Alternating-sign-temperature-shock test (factor: RCWTB content)	Weight	0.0000	-
	UPV	0.0000	*M4.5* and *M6.0*
	Length	0.4655	*M0.0*, *M1.5*, *M3.0*, *M4.5,* and *M6.0*
	Compressive strength	0.0141	-
	Density	0.0000	-
Alternating-sign-temperature-shock test (factor: number of cycles)	Weight	0.0000	-
	UPV	0.0000	-
	Length	0.5018	10 and 20
	Compressive strength	0.0007	-
	Density	0.0000	-
Alternating-sign-temperature-shock test (interaction between factors)	Weight	0.0000	-
	UPV	0.0000	-
	Length	0.4293	*M0.0*, *M1.5*, *M3.0*, *M4.5,* and *M6.0*
	Compressive strength	0.6556	*M0.0*, *M1.5*, *M3.0*, *M4.5,* and *M6.0*
	Density	0.0000	-
High-temperature-shock test (factor: RCWTB content)	UPV	0.0000	-
	Compressive strength	0.0000	-
High-temperature-shock test (factor: duration)	UPV	0.0000	-
	Compressive strength	0.0000	-
High-temperature-shock test (interaction between factors)	UPV	0.0000	-
	Compressive strength	0.0000	-

This statistical analysis showed that the effect of RCWTB on all properties was generally significant, except for the variation of specimen length in the moist/dry and alternating-sign-temperature-shock tests. Thus, it appears that the alteration in the dimensional stability of the concrete was minimal as the content of the recycled waste was increased. In addition, the duration of the alternating-sign-temperature-shock test was not significant in terms of dimensional stability variations, in the same way as the interaction in terms of dimensional stability and compressive strength between both test factors. All factors and the interactions between them had a significant effect on the high-temperature-shock test, which simulated the environmental conditions with the highest effect on the RCWTB concrete.

#### Linear multiple-regression adjustment

Among all the aspects described in this paper, the clearest effect of RCWTB was noted in the high-temperature-shock test: the higher the content of RCWTB, the higher the reduction in concrete compressive strength. In addition, the ANOVA revealed that the decrease in strength with the addition of certain amounts of the waste depended on the test duration, thereby underlining the interaction between both factors (Ma et al. 2023). This interaction is key to the thermal behavior of RCWTB concrete, as it shows the negative effect of RCWTB under high temperatures that is aggravated at longer exposure times.

**Fig. 14:**
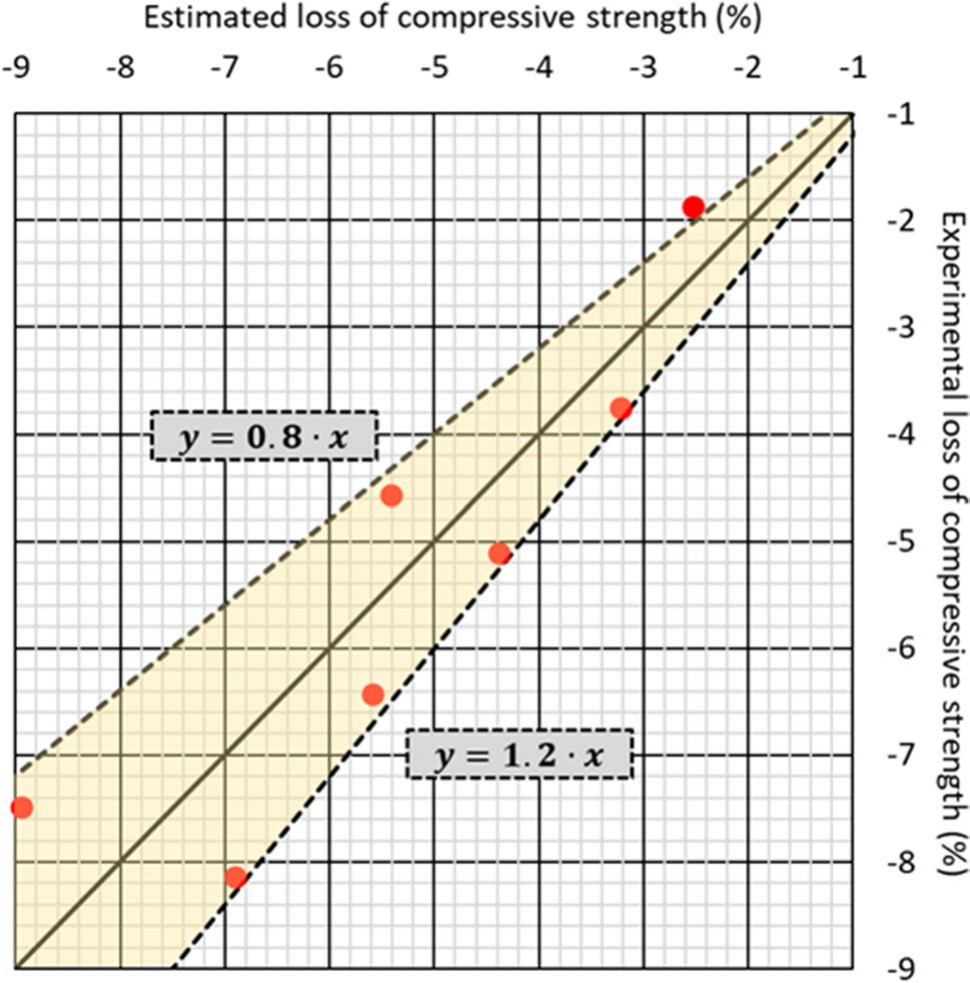
Comparison of experimental and estimated losses of compressive strength during the high-temperature-shock test

A linear multiple-regression model was developed to corroborate the existence of this interaction and to predict the variation of the compressive strength (*∆C*, in %) from the exposure time (*t*, in days) at high temperatures, in this case 200 ± 5 °C, and the RCWTB content of the concrete (*c*_*RCWTB*_, in %). This model is detailed in Eq. (1), which presented an *R*^2^ coefficient of 84.24%. It can be noted that the prediction of the effect of thermal exposure on the RCWTB concrete depended not only on the two aforementioned factors but also on the interaction between them, as shown by the last term of Eq. (1). It shows that the behavior of the RCWTB when exposed to high temperatures can be predicted by a simple linear model, but that any modification of the effect of this waste with the variation of the exposure times must also be considered (Revilla-Cuesta et al. 2021). If those factors are considered, then the model developed in this case can be sufficiently accurate at estimating compressive-strength decreases over 1% with a maximum deviation of ± 20%, as shown in Fig. 14.

1 $$\Delta CS=-0.191-0.083\times t-0.431\times {c}_{RCWTB}-0.133\times t\times {c}_{RCWTB}$$

### Environmental analysis

**Fig. 15:**
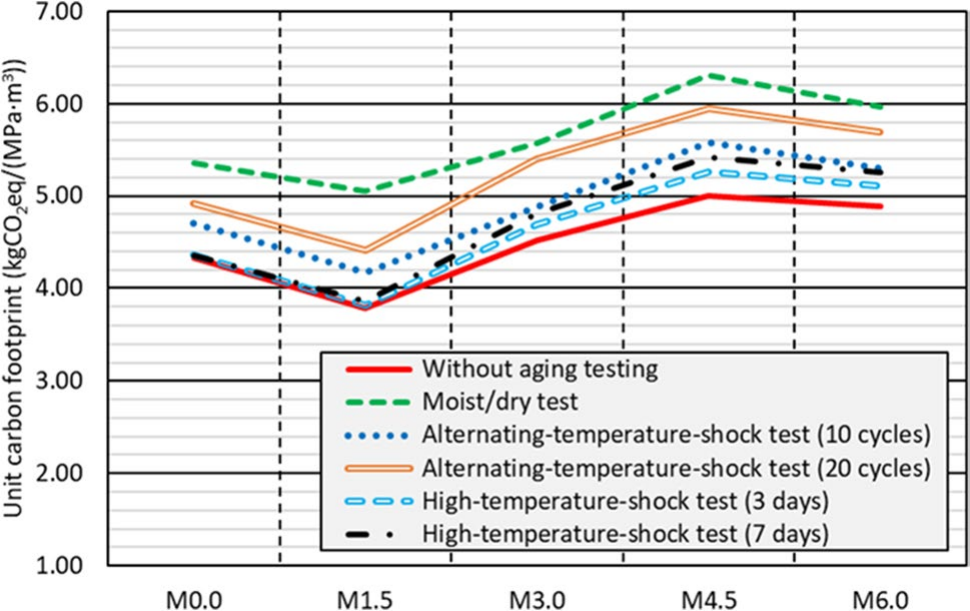
Carbon footprint per unit of compressive strength after each test

The carbon footprint per m^3^ of all the concrete mixes (*CF*_*c*_, in kgCO_2_eq/m^3^) was calculated according to Eq. (2). In this formula, *CF*_*rm*_ is the carbon footprint of each raw material in kgCO_2_eq/kg, obtained from the available scientific literature (Yang et al. 2015; Hossain et al. 2016; Rebello et al. 2019; Revilla-Cuesta et al. 2024), and *Q* is the content of each raw material in the concrete in kg/m^3^, values that are depicted in Table 2. The carbon footprint was always reduced when increasing the RCWTB content (Revilla-Cuesta et al. 2024). Based on this, the carbon footprint per unit of compressive strength of the concrete mixes (*CF*_*c,CS*_, in kgCO_2_eq/(MPa·m^3^)) was obtained through Eq. (3), in which *CS* is the compressive strength in MPa (Hossein et al. 2022). The residual compressive strength after testing was used in this research. Figure 15 shows the unit carbon footprint for each test and mix.

2 $${CF}_{c}=\sum {CF}_{rm}\times Q$$

3 $${CF}_{c,CS}=\frac{{CF}_{c}}{CS}$$

The unit carbon footprint followed a similar trend in all the tests, decreasing when 1.5% RCWTB was added. This was possible due to the decrease in cement content and the improvement of the concrete behavior under moisture and temperature fluctuations thanks to the GFRP-composite fibers (G. T. Xu et al. 2022a, b; Hamada et al. 2023). The increased content of balsa-wood and polyurethane particles with the addition of higher percentages of RCWTB led to a larger loss of compressive strength after testing, so that the *M0.0* and *M3.0* mixes had the same unit carbon footprint, which was even higher in the *M4.5* mix, despite the decrease of cement content per m^3^ in the concrete. The bridging effect of the GFRP-composite fibers was more successful in the *M6.0* mix, which led to a reduction in the unit carbon footprint. Finally, concrete exhibited a better environmental performance when exclusively exposed to high temperatures, as the combination of high temperatures and large moisture variations caused the highest deterioration of compressive strength.

## Conclusions

RCWTB is a waste material from the non-selective crushing of wind-turbine blades, i.e., without prior separation of their components. Its use as a global addition in concrete has the key advantages of GFRP-composite fibers, but it also introduces balsa-wood and polyurethane particles within the concrete, which are in principle sensitive to fluctuating humidity and temperature. Therefore, the behavior of a concrete with up to 6% RCWTB as an overall addition to moisture and temperature changes has been analyzed in this paper by simulating them through moist/dry, alternating-sign-temperature-shock, and high-temperature-shock tests. The following conclusions can be drawn from the results of these tests:

- The application of cyclic moisture and thermal variations (moist/dry and alternating-sign-temperature-shock tests) caused micro-cracking in the cementitious matrix of concrete. This micro-cracking was conditioned by the porosity of the concrete mix. Thus, the micro-cracking within the concrete throughout these tests was greater the higher its porosity, which meant that increasing the RCWTB content of the concrete was not always associated with increased micro-cracking, as the addition of this waste did not necessarily lead to an increase in concrete porosity.
- The cyclic application of moisture and temperature variations also caused deterioration of the RCWTB components within the concrete, which was evident in the lower ultrasonic pulse velocity (UPV) readings throughout the test. The higher the RCWTB amount, the lower the UPV readings, due to the higher proportions of GFRP-composite fibers, polyurethane, and balsa wood within the concrete.
- The application of sub-zero temperatures (− 20 °C) and high-temperature moisture conditions (+ 70 °C) in the alternating-sign-temperature-shock test caused micro-cracking and the deterioration of the RCWTB components to occur earlier than when applying moisture and drying cycles at less extreme temperatures, + 20 °C and + 70 °C, respectively. So, there was no noticeable increase in damage to the RCWTB concrete with the number of cycles in the alternating-sign-temperature-shock test, as the damage mainly occurred in the first few cycles of the test.
- Both micro-cracking and damage to the RCWTB components caused the appearance of remaining strain in the concrete after the tests. Any remaining strain was higher whenever the RCWTB content was higher, and three times greater after the alternating-sign-temperature-shock test, possibly due to the application of sub-zero temperatures. However, the length variation was less than 0.10% in all cases.
- The compressive strength of the concrete decreased due to both micro-cracking and damage to RCWTB components. However, this reduction in strength in both the moist/dry and alternating-sign-temperature-shock tests was globally greater for mixes containing 1.5–3.0% RCWTB than for those with higher RCWTB contents, for which the strength decrease stabilized. Although they could be damaged during the tests due to the thermal fluctuations, GFRP-composite fibers could also effectively maintain their bridging effect within the cementitious matrix after the tests, thereby compensating the greater deterioration of the balsa-wood and polyurethane particles following the addition of RCWTB in large amounts.
- Exposure of RCWTB concrete to sustained high temperatures (high-temperature-shock test) clearly revealed the mode of deterioration of both the polyurethane and the balsa-wood particles during these tests. The polyurethane particles melted, while the balsa-wood particles burned. Those reactions increased concrete porosity and weakened the interfacial transition zones; so, the higher the RCWTB content, the higher the losses of compressive strength. However, the bridging effect of the GFRP-composite fibers was maintained when 6.0% RCWTB was added.
- Statistical analyses of the test results showed that the added content of RCWTB significantly affected the response of concrete exposed to moisture and temperature fluctuations. The interaction of RCWTB content and exposure time to moisture and high temperatures also played a fundamental role in predicting the behavior of RCWTB concrete under those conditions, as the effects of different RCWTB contents varied according to the exposure time.

Overall, the performance of concrete containing RCWTB under temperature and humidity variations and high temperatures was suitable regarding the features analyzed in this paper. Furthermore, this waste improved concrete sustainability. Nevertheless, precise estimation of the exposure time is essential, in order to define adequate amounts of the RCWTB that will ensure proper behavior of concrete in real applications in which concrete is exposed to such environmental conditions.

## Limitations of the study and future research lines

Notwithstanding the conclusions reached, the study presents different limitations that should be noted and addressed in future research aimed at utilizing concrete with RCWTB in applications exposed to high temperatures or variations in temperature and humidity. They are detailed below:

- First, further research regarding the bending behavior of concrete containing RCWTB after exposure to simultaneous variations in humidity and temperature and high temperatures could be useful. The objective should be to evaluate the effectiveness of the bridging effect of GFRP-composite fibers under such environmental conditions. This performance should be analyzed both in specimens and in full-scale structural elements, such as slabs or beams, to simulate real applications.
- Second, balsa wood and polyurethane are the most sensitive RCWTB components to temperature and humidity variations and high-temperature exposure, so they suffer the greatest deterioration. Therefore, tests precisely only on these RCWTB components should be conducted. Furthermore, possible surface treatments of balsa wood to improve its performance should be examined.
- Third, the fire resistance of concrete with RCWTB should be further investigated, since balsa-wood and polyurethane particles can be very sensitive to these exposure conditions.
- Finally, the durability performance of RCWTB concrete in other environmental conditions, such as freeze/thaw, marine environment, or gas-rich industrial atmospheres, should be evaluated. In this way, the range of applications of RCWTB concrete could be extended.

## Data Availability

All the data generated in this research are contained within the article.
